# The bone attachments of the medial collateral and posterior oblique ligaments are defined anatomically and radiographically

**DOI:** 10.1007/s00167-020-06139-6

**Published:** 2020-07-31

**Authors:** K. K. Athwal, L. Willinger, S. Shinohara, S. Ball, A. Williams, Andrew A. Amis

**Affiliations:** 1 The Biomechanics Group, Department of Mechanical Engineering, Imperial College London, London, SW7 2AZ UK; 2 Department of Orthopaedic Sports Medicine, Klinikum rechts der Isar, Technical University Munich, Munich, Germany; 3 Faculty of Engineering, Hokkaido University, Sapporo, 060-8628 Japan; 4 Fortius Clinic, 17 Fitzhardinge St, London, W1H 6EQ UK; 5 Musculoskeletal Surgery Group, Imperial College London School of Medicine, London, W6 8RF UK

**Keywords:** Medial collateral ligament, Posterior oblique ligament, Bone insertion attachment footprint, Knee anatomy, Radiograph

## Abstract

**Purpose:**

To define the bony attachments of the medial ligaments relative to anatomical and radiographic bony landmarks, providing information for medial collateral ligament (MCL) surgery.

**Method:**

The femoral and tibial attachments of the superficial MCL (sMCL), deep MCL (dMCL) and posterior oblique ligament (POL), plus the medial epicondyle (ME) were defined by radiopaque staples in 22 knees. These were measured radiographically and optically; the precision was calculated and data normalised to the sizes of the condyles. Femoral locations were referenced to the ME and to Blumensaat’s line and the posterior cortex.

**Results:**

The femoral sMCL attachment enveloped the ME, centred 1 mm proximal to it, at 37 ± 2 mm (normalised at 53 ± 2%) posterior to the most-anterior condyle border. The femoral dMCL attachment was 6 mm (8%) distal and 5 mm (7%) posterior to the ME. The femoral POL attachment was 4 mm (5%) proximal and 11 mm (15%) posterior to the ME. The tibial sMCL attachment spread from 42 to 71 mm (81–137% of A-P plateau width) below the tibial plateau. The dMCL fanned out anterodistally to a wide tibial attachment 8 mm below the plateau and between 17 and 39 mm (33–76%) A-P. The POL attached 5 mm below the plateau, posterior to the dMCL. The 95% CI intra-observer was ± 0.6 mm, inter-observer ± 1.3 mm for digitisation. The inter-observer ICC for radiographs was 0.922.

**Conclusion:**

The bone attachments of the medial knee ligaments are located in relation to knee dimensions and osseous landmarks. These data facilitate repairs and reconstructions that can restore physiological laxity and stability patterns across the arc of knee flexion.

## Introduction

The superficial medial collateral ligament (sMCL), the deep medial collateral ligament (dMCL) and the posterior oblique ligament (POL)—a part of the posteromedial capsule (PMC)—are the medial ligamentous stabilisers of the knee against valgus and rotatory loads [2, 8, 10, 24, 32]. The MCL is the most frequently injured ligament of the knee [1] and can mostly be treated non-surgically with good clinical results [6, 12, 14, 23]. However, surgery is indicated in high grade MCL injuries and also when valgus instability persists in spite of conservative treatment [7, 11, 16, 19, 22] and with lesser degrees of laxity in combined ligament injury.

Orthopedic surgeons rely on the definition of the ligament attachment points to be able to perform and evaluate operations accurately. Inaccurate MCL reinsertion or graft tunnel placement would either cause ligament over-tension and over-constraint of the knee, or an insufficient and loose reconstruction, across the arc of knee motion [3, 35]. There are significant differences of isometry, or length-change behaviour, across the widths of each of the medial ligaments [34], so it is apparent that accurate positioning is important if the normal patterns of restraint are to be reproduced.

Medial knee anatomy has been described using several different methods. Besides anatomical dissection [20, 21, 25, 31], lateral radiographs [33] and computed tomography [26] have also been used to define the soft-tissue attachment points. Despite many previous publications, conflicting anatomical descriptions still exist. While many anatomical studies have found the femoral sMCL attachment on the medial epicondyle (ME) [13, 21, 25, 36], which would seem logical given that bony prominences usually correspond to soft tissue attachment sites, it has been described as completely separate, proximal, and posterior [20]. Definitions of the attachment points in relation to different bony landmarks given as absolute dimensions in mm do not account for the large range of sizes of the femoral condyle and proximal tibia across the population, so it is desirable to have normalised data. Furthermore, and surprisingly, despite its important function to restrain tibial external rotation [2, 4, 24], the attachment points of the dMCL have only ever been reported by Liu et al. [21] and Robinson et al. [25]. These differences and limitations demonstrate the need for more definitive guidance for the surgeon, such as where to position tunnels for anatomical MCL grafts, or to aid critical evaluation of those positions achieved in images post-surgery as quality control.

The purpose of this study was to provide measurements to define the medial ligament complex (that is: sMCL, dMCL and POL) bony attachments (‘footprints’) on the femur and tibia using both optical navigation and radiographic methods. These objective anatomical and radiographical data are described for the first time both in relation to useful surgical bony landmarks, and also normalised in relation to the overall sizes of the bones. These data will facilitate anatomical repairs and reconstructions that can restore physiological laxity and stability patterns across the arc of knee flexion.

## Materials and methods

Following approval from the Imperial College Healthcare Tissue Bank, Human Tissues Authority licence 12275, application R18027, 22 non-paired fresh-frozen human cadaveric knees (15 male and 7 female) with an average age of 47 (range 24–69) years were used. All specimens were stored at − 20 °C and thawed for 24–36 h before use. All knees were free of osteoarthritis and ligaments and menisci were intact; this was confirmed by inspection during the dissection process. Knees were kept moist with intermittent water spray during the entire test.

The femur and tibia were cut 200 mm from the joint line in the first 12 knees, then at 200 and 150 mm respectively in the remaining 10 knees. The fibula was cut and secured to the tibia in its anatomical position by a transcortical bone screw. Skin, subcutaneous fat, muscles, and the anterior capsule with the patella were removed, keeping the cruciate and collateral ligaments and the remaining capsule intact. Intramedullary (IM) rods were cemented into the tibia and femur to allow subsequent mounting in adjustable clamps.

In the first 12 knees a metal pin 1 mm diameter was inserted into the bone at the point judged visually and by palpation to be the femoral ME to allow radiographic measurement of its position.

**Fig. 1:**
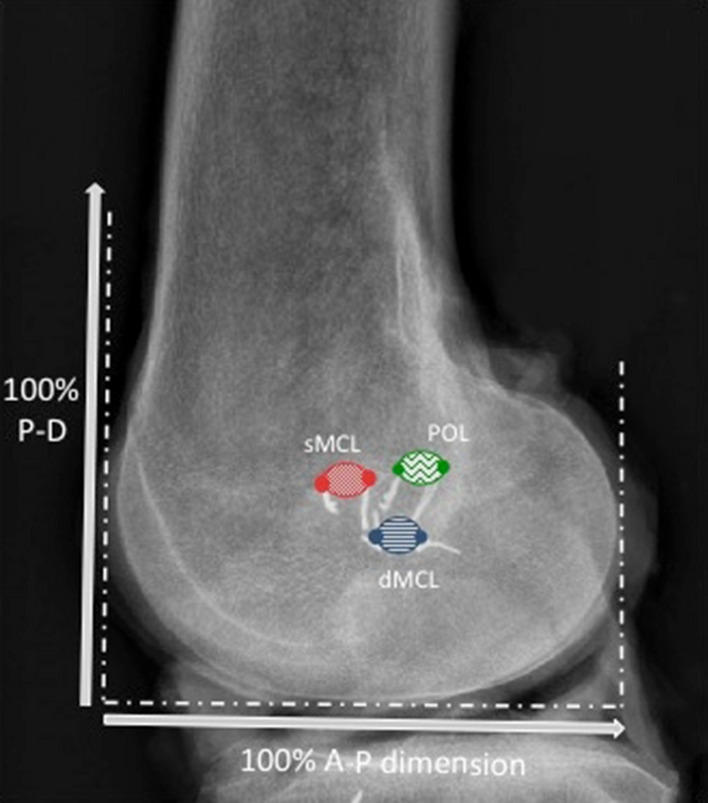
Femoral attachments of the superficial MCL (sMCL), deep MCL (dMCL) and the posterior oblique ligament (POL) as seen radiographically, in relation to the normalisation as a percentage of the AP size of the medial femoral condyle. Note that the wire staples were inserted into the bone with the fold in the staple at the point of interest, and the radiographic study required identification of which end of each staple was the correct measurement point at the surface of the bone (coloured areas). The P-D measurements use the same 100% normalising length as the A-P measurements

**Fig. 2:**
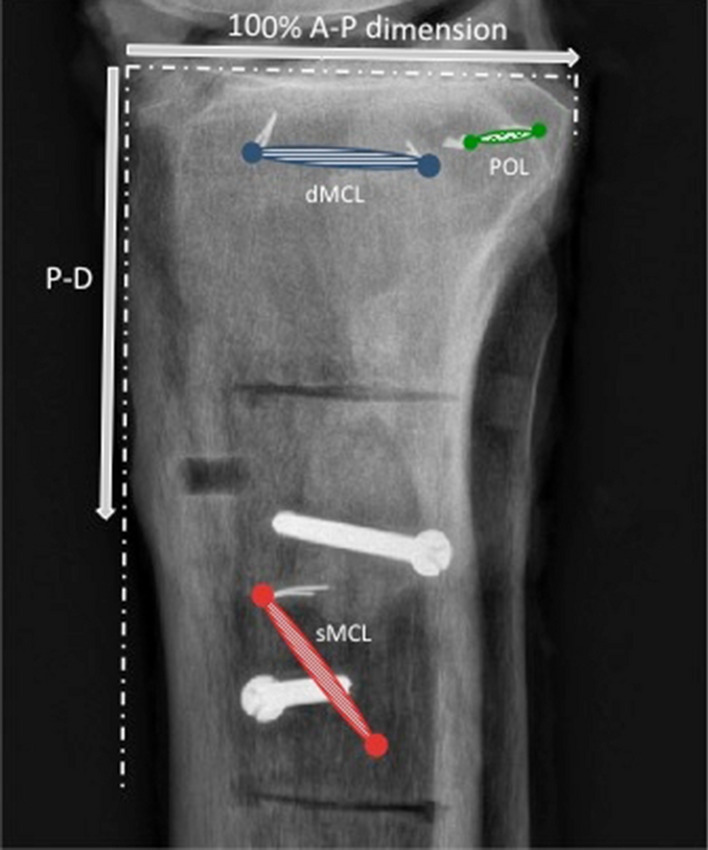
Tibial attachments of the superficial MCL (sMCL), deep MCL (dMCL) and the posterior oblique ligament (POL) as seen radiographically, in relation to the normalisation as a percentage of the A-P size (100%) of the medial tibial plateau, in both A-P and P-D directions. The arrow related to P-D measurement uses the same 100% length as the A-P measurements. Saw cuts for the elevation of a block of bone with the distal attachment of sMCL can be seen. The 2 distal screws have been used to fix the bone block back in place

In the remaining 10 knees the sartorius fascia (layer 1 of Warren and Marshall [31]) along with the semitendinosus and gracilis tendons were removed from their tibial attachments to visualise the sMCL and POL within the second medial soft tissue layer. The connecting fibres between the POL and the semimembranosus tendon were dissected to uncover the distal POL on the tibia proximal to the semimembranosus groove. The behaviour of the sMCL and POL fibres was observed through the range of knee motion to identify the border between the sMCL and POL. The femoral and distal tibial attachments of the sMCL were identified and each was defined by two radiopaque metal staples (6 mm long × 1 mm wide); these markers were inserted into the bone at the most anterior and posterior edges of each attachment (Figs. 1, 2). The attachments of the POL were likewise marked with staples at the most anterior and posterior edges of the femoral and tibial attachments. A tibial bone block (average 45 mm length × 28 mm width × 14 mm depth) including the tibial sMCL attachment was created, elevated and reflected en bloc proximally by sharp dissection along the margins of the overlying sMCL to expose the dMCL. Staples were inserted at the anterior and posterior edges of the femoral and tibial attachments of the dMCL. The tibial bone block was then replaced and fixed with two bi-cortical bone screws placed in pre-drilled holes over a 1 mm thick spacer to compensate for the saw cut bone loss. This held the distal sMCL in its anatomical relationship for digitisation and radiography. Using a linear displacement transducer it was confirmed by measurement that this procedure did not alter the length of the sMCL fibres significantly (≤ 0.5%) [34].

### Digitisation of attachments

The 10 knees with metal staples were each clamped on a measurement table with the intramedullary rods held in adjustable stands at 0° flexion, with the medial aspect of the knee facing upwards. The femoral posterior condylar axis was adjusted to be perpendicular to the table using a template. The anterior and posterior edges of the femoral and tibial attachments of the ligaments were taken as the looped ends of the staples that were flush with the cortex. These and other anatomical landmarks, such as the ME, the adductor and gastrocnemius tubercles, and the proximal and distal edges of the ligament attachments, were digitised using an optical tracker stylus probe with reflective markers (BrainLab AG, Germany). The position of the probe was tracked by an optical tracking system (Polaris Vega, Northern Digital Inc, Canada), which had a volumetric root mean square error of 0.12 mm, and a custom MATLAB (MathWorks, Natick, MA) script was then used to determine the positions of the attachment points relative to anatomical landmarks in the coordinate system of Grood and Suntay [9].

In order to calculate both the intra- and inter-observer precision of the digitising of anatomical landmarks without metal markers the ME of two knees were each located eleven times each by three blinded observers (*n* = 66). The precision of digitising the metal markers was evaluated by two observers making four sets of measurements, and repeated on two occasions. Descriptive statistics including 95% confidence intervals (CI), and 95% prediction intervals were calculated in both mm and as normalised  % of the A-P size of the femoral medial condyle.

### Medial–lateral radiographs

The knees were imaged radiographically in a true medial–lateral orientation—verified by superimposing the posterior femoral condyles on the radiographs—at 0° flexion, with the intramedullary rods clamped as above. A radiopaque ball 25 mm diameter and a radiography ruler held at the level of the medial femoral condyle were captured on each image to allow correction of magnification. The images were analysed by two examiners (blinded) using Clarity Viewer (Condonics, Ohio, US) software at a mean magnification of ×3.3.

### Normalised results and coordinate system

With each imaging technique, a two-dimensional sagittal plane coordinate system was created to locate the attachment points on the femur and tibia. Datum points were defined in order to allow normalisation for the overall size of each bone. The datum points of the distal femur were the most anterior, posterior, and distal points of the medial femoral condyle (MFC) (Fig. 1). The datum points of the proximal tibia were the anterior and posterior edges of the medial tibial plateau (MTP) (defined in Fig. 2). The location of each femoral attachment was normalised to the A-P dimension of the MFC (= 100%) and also referenced to the ME, while the tibial attachments were normalised to the A-P dimension of the MTP (= 100%). The A-P sizes of the femoral and tibial condyles were also used to define the P-D (proximal–distal) dimensions. All data were recorded as actual measurements in mm, then normalised to the size of the bone in percentages.

The optical tracker measurements were cross-checked versus the radiographs in order to ensure that the correct end of each metal staple (at the bone surface) was identified radiographically.

**Fig. 3:**
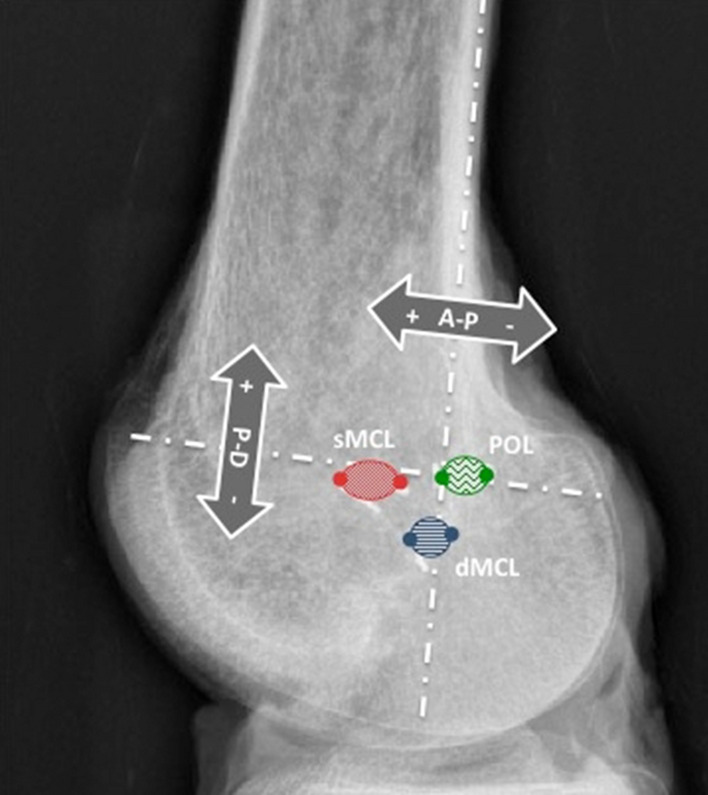
Method to define positions radiographically, using datum lines that (1) form an extension of the line of the posterior femoral cortex, and (2) a line perpendicular to the former passing through the most-posterior/proximal point of Blumensaat’s line [27]

A system for radiographic location of femoral attachment points that is easy to use in the clinic was defined by Schöttle et al. [27]: the points were referenced to a line extending distally from the posterior femoral cortex and to a perpendicular line intersecting the most-posterior/proximal edge of Blumensaat’s line (Fig. 3). The distances were measured perpendicular to the reference lines in A-P and P-D directions, as actual sizes in mm and then were normalised.

### Statistical analysis

Data were analysed using SPSS version 24 (SPSS Inc, Chicago, IL) to determine variability within and across the optical tracker and radiographic methods. With sets of 11 specimens, it was determined that a measurement SD = 0.7 mm would yield a 95% CI of the mean of ± 0.2 mm. The anatomical measurements of each method were given in mean ± SD. Intra-class correlation coefficients (ICC) were calculated on the measured A-P widths of the femora (MFC) and tibiae (MTP) to find: inter-observer reliability of two blinded examiners when analysing radiographs; test–retest reliability of digitising attachment points with a stylus probe with four examiners; and test comparison reproducibility across radiographic and digitising techniques using four repeated measurements. ICC values between 0.5 and 0.75 were considered moderate, between 0.75 and 0.9 considered good, and greater than 0.90 were considered excellent reliability [5, 18].

## Results

The test–retest reliability of the optical digitisation technique had an ICC of 0.994 (excellent agreement). When 3 examiners digitised the ME the mean intra-observer 95% CI of the mean was ± 0.6 mm in A-P and ± 0.5 mm in P-D directions, while the mean inter-observer differences were ± 1.8 mm in A-P and ± 0.9 mm in P-D directions. The mean intra-observer difference of radiographic measurements was 0.8%, equivalent to 0.5 mm across the width of the femoral condyle. The inter-observer reliability test of the radiographic analysis between two blinded examiners gave ICC 0.922—excellent agreement.

### Bone dimensions

The mean A-P width of the MFC (= 100% for normalisation) was 69 ± 5 mm (range 59–76 mm) and the MTP was 52 ± 5 mm (range 43–58 mm).

### Location of the medial femoral epicondyle

**Fig. 4:**
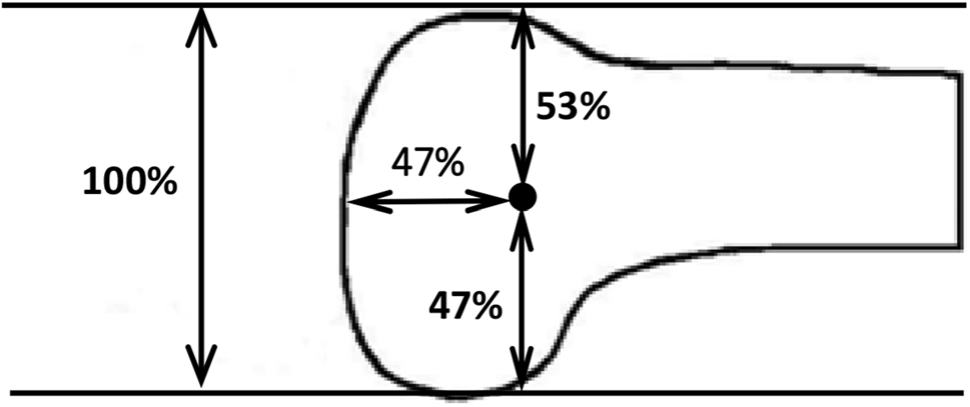
The femoral medial epicondyle, which was also the centre of the sMCL femoral attachment (marked as the black dot), was normalised in relation to the size of the medial femoral condyle: if mean A-P size of 69 mm was 100%, then the epicondyle was: 47% (32 mm) from both the posterior and distal and 53% (37 mm) from the anterior outline

The ME was 53 ± 2% (37 mm mean) posterior to the most anterior point of the MFC and 47 ± 2% (32 mm mean) superiorly from the most distal point and the same distance anterior from the most posterior point (Fig. 4).

### Bone attachments of the sMCL

**Fig. 5:**
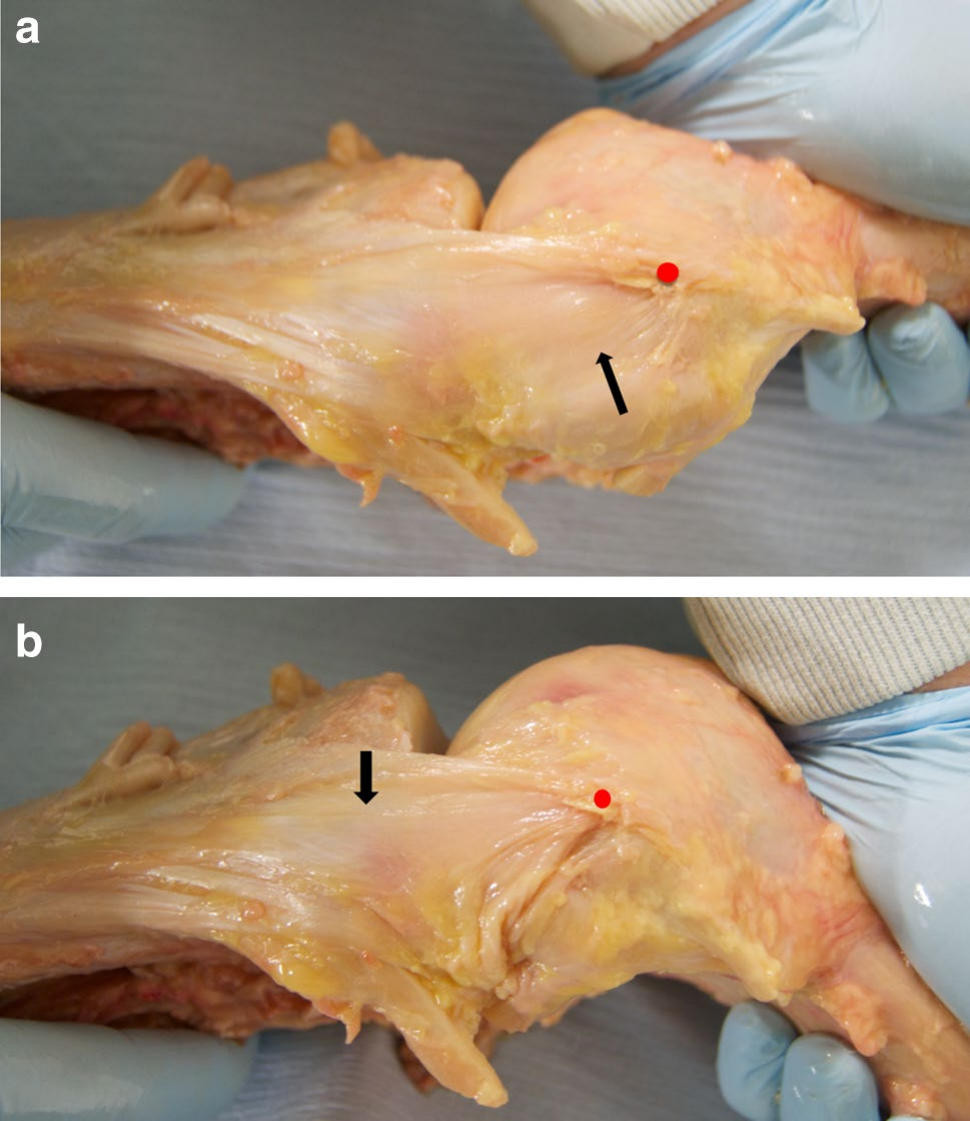
**a** The sMCL and PMC are shown with the knee in full extension. The medial epicondyle is at the red dot. The PMC/POL is taut with the knee in full extension (black arrow). **b** The sMCL and PMC/POL are shown with the knee flexed. The sMCL fibres (black arrow) centred their femoral attachment onto the medial epicondyle (red dot) and remained taut with knee flexion. The PMC/POL is slack with the knee flexed

**Fig. 6:**
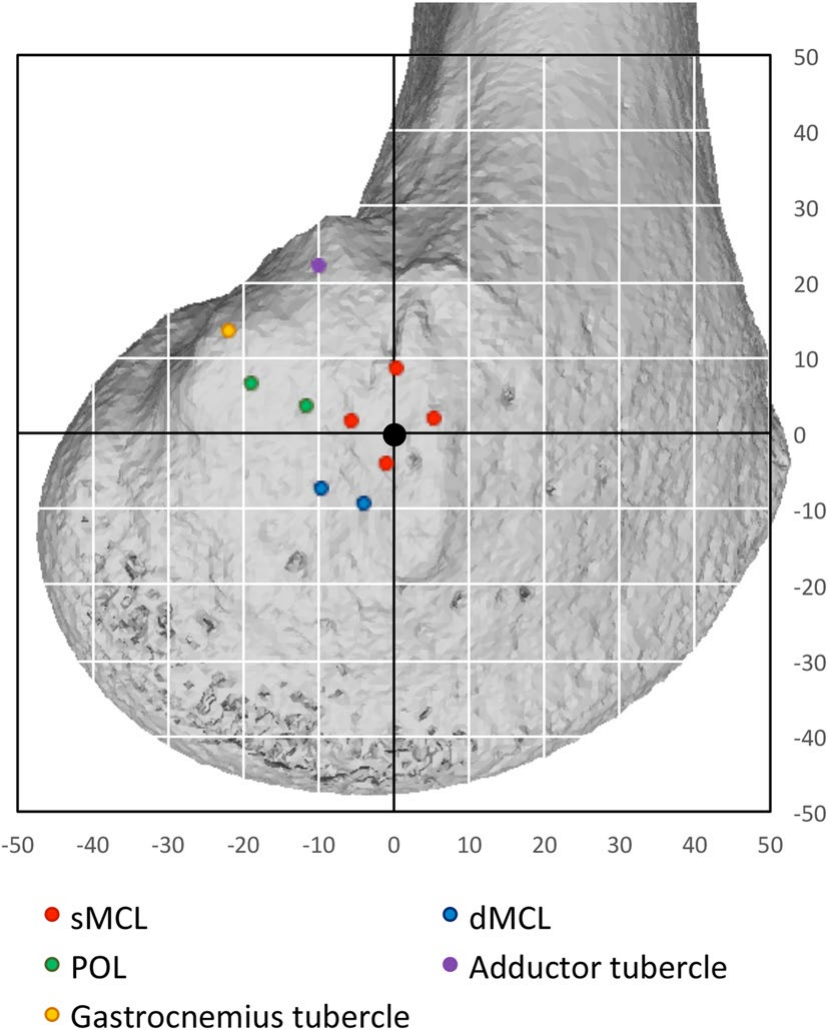
Mean limits of the soft tissue attachments relative to the medial epicondyle (ME-black dot) in a left knee as measured by optical digitisation, normalised to the size of the medial femoral condyle (MFC). The grid is offset so that it is centred at the ME at 53% posterior and 47% proximal to the edges of the MFC, as in Fig. 4. Data are superimposed onto a representative CT reconstruction. Data from the radiographic analysis (Table 1) were effectively the same when drawn in this manner, so are not shown

The femoral attachment of the sMCL always covered the ME (Fig. 5a, b). It was 7 mm wide (11% of the condyle dimension) in A-P (Table 1) and 9 mm wide (13%) P-D, centred 1–2 mm proximal to the ME (Fig. 6).

**Table 1:** Normalised distance (% of AP medial femoral condyle width where 100% = 69 ± 5 (59-–6) mm) of the femoral attachment points of the soft-tissues relative to the medial epicondyle, using both digitisation with optical tracking and radiography (mean ± standard deviation, *n* = 10)

	Anterior sMCL	Posterior sMCL	Anterior dMCL	Posterior dMCL	Anterior POL	Posterior POL
Digitisation						
AP	5 ± 1	− 6 ± 2	− 4 ± 3	− 10 ± 3	− 12 ± 3	− 19 ± 4
PD	2 ± 1	2 ± 1	− 9 ± 3	− 7 ± 3	4 ± 1	7 ± 3
Radiographs						
AP	6 ± 2	− 5 ± 2	− 4 ± 4	− 11 ± 4	− 13 ± 3	− 20 ± 4
PD	1 ± 3	2 ± 3	− 10 ± 3	− 8 ± 3	3 ± 3	5 ± 4

Key: *AP* anterior–posterior direction, with positive values indicating attachment points anterior to the epicondyle. *PD* proximal–distal direction, with positive values indicating attachment points proximal to the epicondyle. *sMCL* superficial medial collateral ligament. *dMCL* deep medial collateral ligament. *POL* posterior oblique ligament

**Fig. 7:**
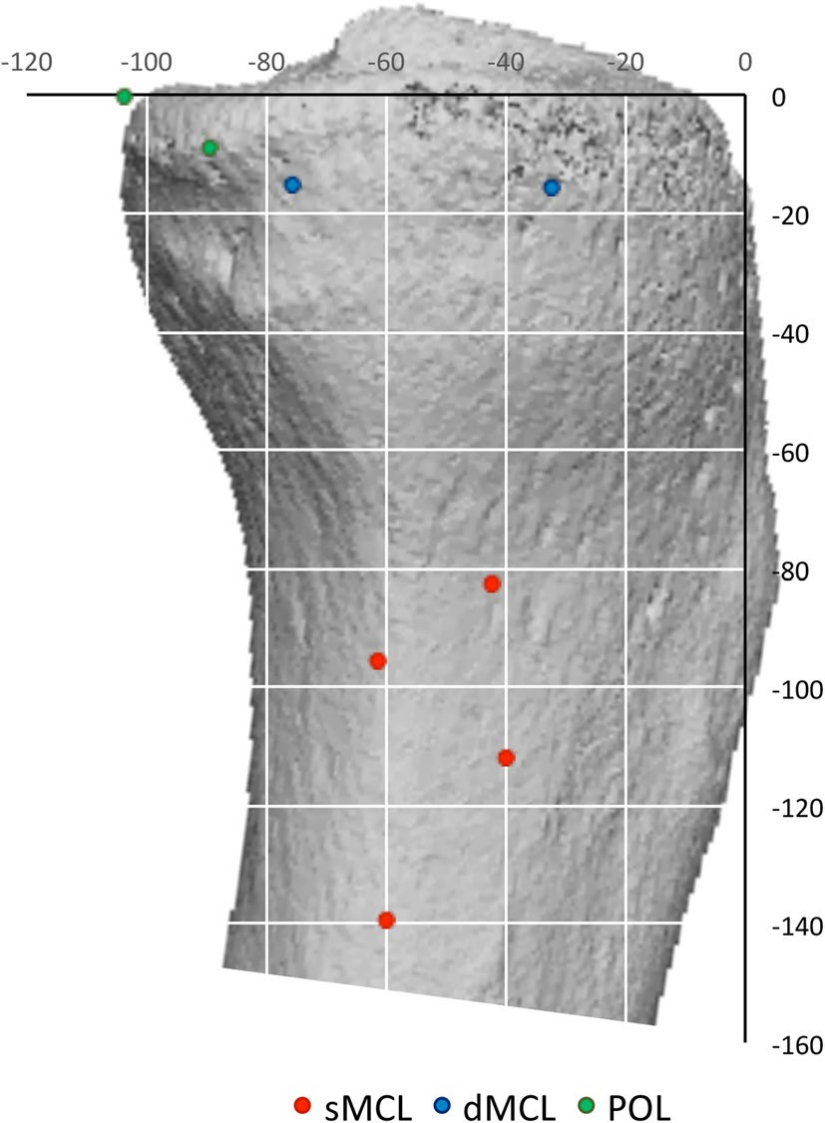
Mean tibial attachment points of the superficial medial collateral ligament (sMCL), deep medial collateral ligament (dMCL) and posterior oblique ligament (POL) from optical digitisation measurements. Data are superimposed onto a representative CT reconstruction. Data from the radiographic analysis (Table 2) were effectively the same when drawn in this manner, so are not shown

The sMCL coursed directly distally from its femoral attachment to the tibia. The dense distal tibial bony attachment of the sMCL was primarily linear, extending from a mean of 42–71 mm distal to the tibial plateau, (Fig. 7 and Table 2). The sMCL also attached near the proximal tibia, to soft tissue overlying the semimembranosus tendon. It was not to bone as has previously been reported [20] and was flimsy and easily broken down.

**Table 2:** Normalised distance (% of AP medial tibial plateau width where 100% = 52 ± 5 (43–58) mm) of the tibial attachment points of the soft-tissues relative to the most anterior edge of the medial tibial plateau, using both digitisation with optical tracking and radiography (mean ± standard deviation, *n* = 10)

	Anterior sMCL	Posterior sMCL	Anterior dMCL	Posterior dMCL	Anterior PMC	Posterior PMC
Digitization						
AP	40 ± 9	60 ± 11	33 ± 9	76 ± 9	89 ± 13	104 ± 9
PD	112 ± 12	140 ± 21	16 ± 4	15 ± 4	9 ± 5	0 ± 5
Radiographs						
AP	35 ± 3	58 ± 8	28 ± 7	69 ± 6	84 ± 6	99 ± 2
PD	111 ± 11	138 ± 13	16 ± 4	15 ± 3	10 ± 3	6 ± 4

Key: *AP%* posterior to the anterior edge of the medial tibia plateau. *PD* proximal–distal to the medial tibial plateau. *sMCL* superficial medial collateral ligament. *dMCL* deep medial collateral ligament. *POL* posterior oblique ligament

### Bone attachments of the dMCL

**Fig. 8:**
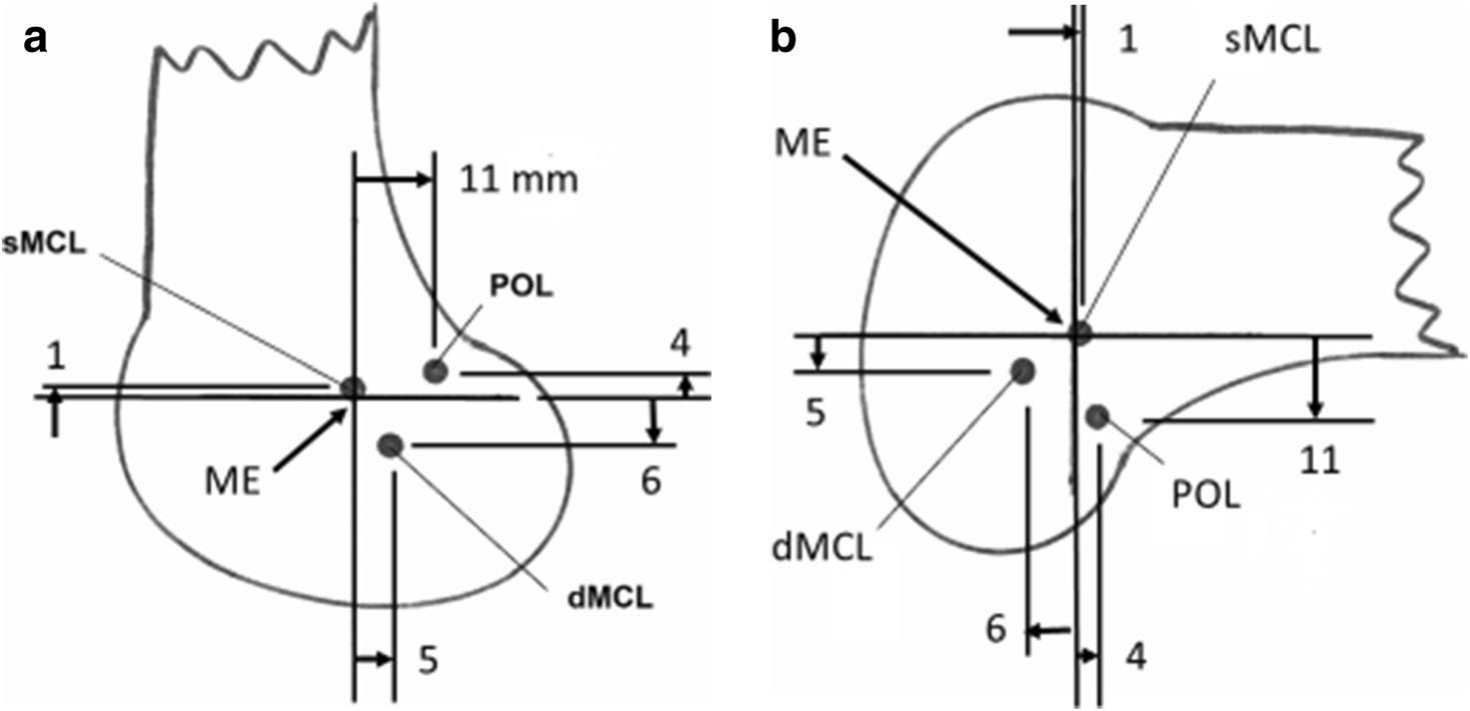
The mean positions (mm) of the centres of the ligament attachments relative to the medial epicondyle (ME). *sMCL* superficial medial collateral ligament, *dMCL* deep medial collateral ligament, *POL* posterior oblique ligament. **a** Anatomic orientation; **b** oriented as in surgery

**Fig. 9:**
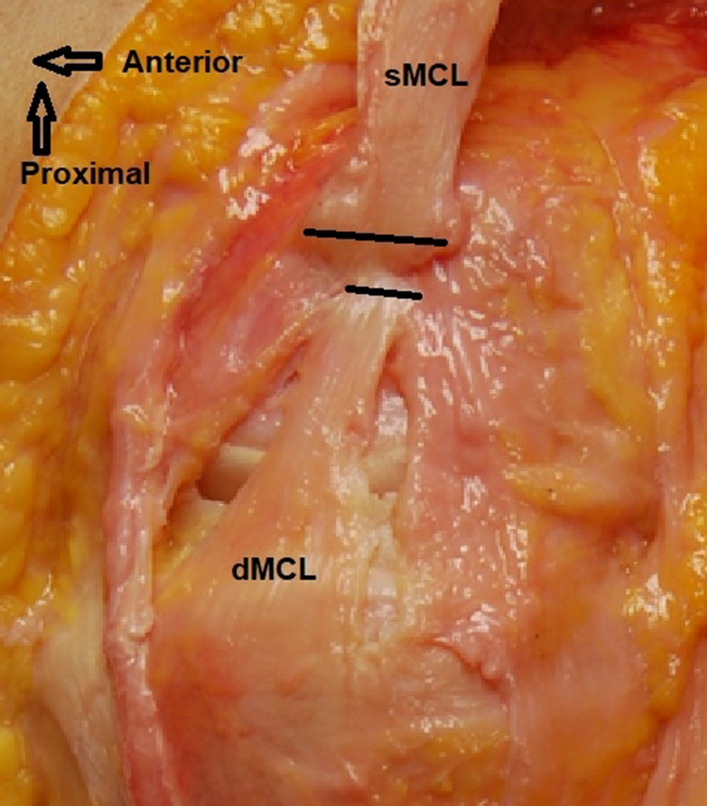
Medial aspect of a right knee in extension. The superficial medial collateral ligament (sMCL) has been elevated from distal (at the bottom of the picture) to proximal to display the deep medial collateral ligament (dMCL). The joint capsule has been split alongside the edges of the dMCL, revealing the femoral condyle. The lines across the femoral attachments of the ligaments show their A-P widths. The midpoint of the attachment of the dMCL is distal and posterior to the midpoint of the attachment of the sMCL. The fibres of the dMCL fan out distally and anteriorly to give a wide tibial attachment, making it ideally aligned to counter tibial external rotation

The femoral attachment of the dMCL was a mean 6 mm (8% of the MFC A-P size) distal to the ME, and so also distal to the sMCL attachment (Fig. 6), with a mean A-P width of 4 mm, and centred a mean 5 mm posterior to the ME (Fig. 8). It was therefore also posterior to the centre of the sMCL attachment (Figs. 8, 9).

**Fig. 10:**
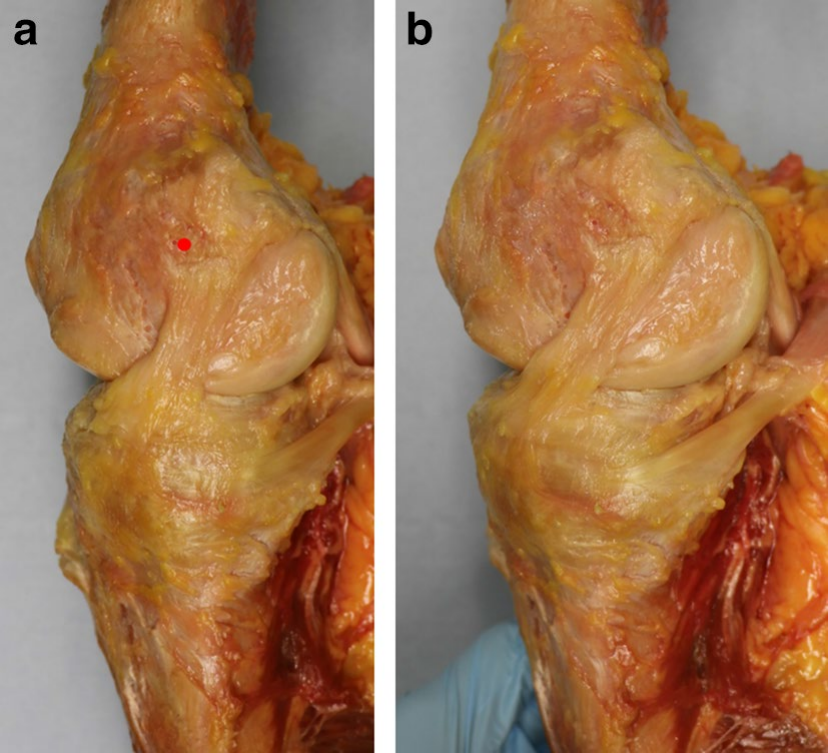
**a** Medial aspect of a right knee. The superficial medial collateral ligament (sMCL) has been excised to display the deep medial collateral ligament (dMCL); the red dot is at the centre of the attachment of the sMCL. The femoral attachment of the dMCL is distal and posterior to the attachment of the sMCL. The joint capsule has been removed anterior and posterior to the edges of the dMCL, revealing the femoral condyle. In neutral tibial rotation the dMCL of this knee was oriented 24° from parallel to the long axis of the tibia at 15° knee flexion. **b** In external tibial rotation the dMCL of this knee was oriented 36° from parallel to the long axis of the tibia at 15° knee flexion

The fibres of the dMCL fan out to a 22 mm mean wide tibial attachment, spreading from 33 to 76% posterior from the anterior edge of the medial plateau, (Figs. 7, 9; Table 2), and was 8 mm mean (15%) distal to the plateau. The dMCL fibres were aligned antero-distally from the femur to the tibia, so it is well aligned to resist tibial external rotation (Fig. 10).

### Bone attachments of the POL

The PMC was continuous with the posterior border of the sMCL in all specimens, at the line of fusion of the posterior edges of the deep and superficial MCL layers [24, 30]. The POL fibres were identified within the expanse of the PMC, oriented postero-distally and tightened by tibial internal rotation near to terminal knee extension. The femoral attachment of the POL was 11 mm posterior and 4 mm proximal to the ME (Figs. 6, 8).

The POL passed (Figs. 5a, 7) to a tibial attachment 7 mm A-P wide on the postero-medial rim of the tibial plateau proximal and also distal to the semimembranosus tendon, thereby creating a tunnel for that tendon that extended beyond the posterior edge of the plateau 100% point. These attachments were 0–5 mm distal to the plateau respectively.

### Radiographic positioning of femoral attachments

**Fig. 11:**
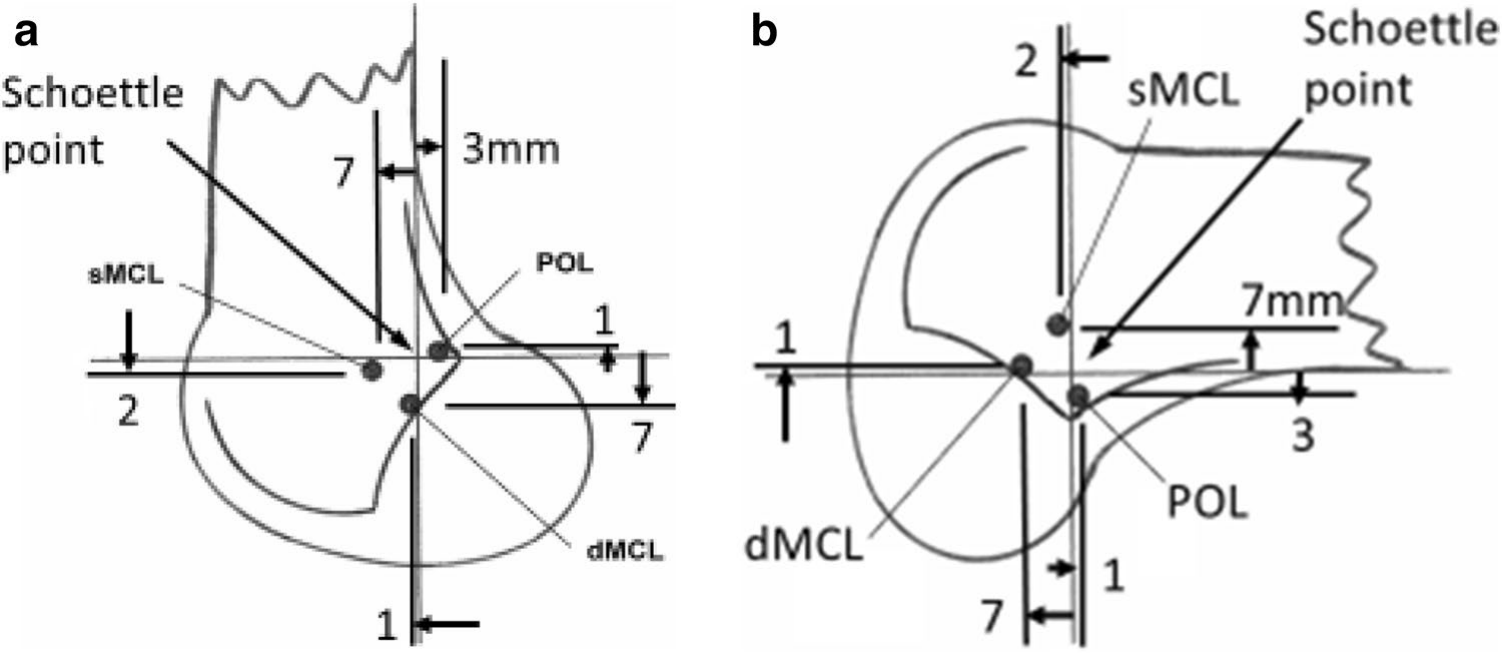
The mean positions (to nearest mm) of the centres of the ligament attachments relative to the Schöttle point [27] at the extended posterior cortex of the femur and the most posterior point of Blumensaat’s line. *sMCL* superficial medial collateral ligament. *dMCL* deep medial collateral ligament, *POL* posterior oblique ligament. **a** Anatomical orientation, and **b** as in surgery

The mean femoral attachments of the sMCL, dMCL and POL relative to Schöttle’s point [27] (Fig. 3) are shown in Fig. 11 and Table 3.

**Table 3:** Distance (mm) and normalised distance (% of AP medial femoral condyle width where 100% = 69 ± 5 (59–76) mm) of the femoral attachments of the medial soft-tissues using Schöttle’s radiographic technique (mean ± standard deviation, *n* = 10)

	Anterior sMCL	Posterior sMCL	Anterior dMCL	Posterior dMCL	Anterior POL	Posterior POL
Length (mm)						
AP	10 ± 3	3 ± 3	3 ± 3	− 1 ± 3	− 1 ± 4	− 5 ± 4
PD	− 2 ± 3	− 1 ± 4	− 8 ± 3	− 7 ± 3	0 ± 4	1 ± 4
Normalised (%)						
AP	16 ± 5	5 ± 6	5 ± 6	− 2 ± 7	− 2 ± 8	− 9 ± 8
PD	− 3 ± 6	− 2 ± 6	− 13 ± 6	− 11 ± 5	0 ± 6	2 ± 6

Key: *AP* anterior–posterior direction, with positive values indicating attachment points anterior to a line along the posterior femoral cortex. *PD* proximal–distal direction, with positive values indicating attachment points proximal to an AP line passing through the most-posterior point of Blumensaat’s line. *sMCL* superficial medial collateral ligament. *dMCL* deep medial collateral ligament. *POL* posterior oblique ligament

## Discussion

The most important outcome of this study is a quantitative anatomical description of the attachments of the medial knee ligaments in relation to the femoral ME and the tibial plateau, and radiological positions with respect to the ‘Schöttle point’ [27]. This knowledge is clinically very useful and practical to use during surgery. The normalised data allow us to relate the results to any knee size: the range of sizes of knees limits the value of descriptions of attachment sites with only mean dimensions. The accuracy and repeatability of the methods are demonstrated to be high. It provides a method for intraoperative identification and postoperative evaluation of the anatomical attachments and MCL graft tunnel positions. The isometric patterns of the ligaments, their repairs and reconstructions, depend on the exact femoral attachments, with significant differences even across the widths of each structure [34], so the data in this paper are a foundation for surgery aiming to restore normal knee behaviour.

Previous anatomical descriptions have sometimes been contradictory and do not allow for definite conclusions [13, 20, 21, 25, 26, 31]. Furthermore, whilst accurate intraoperative location of anatomical landmarks on the medial aspect of the knee is important it is often difficult by palpation alone. Therefore, in the operating room landmark identification is often best achieved with a combination of visualisation, palpation and radiographic localisation. This paper used the radiographic method of Schöttle et al. [27] to define the MCL attachments because of the lack of visibility of the ME in lateral view radiographs [33].

The study confirms previous reports [13, 21, 25] that the femoral sMCL attachment covers the ME. It seems logical for the sMCL to attach directly to the ME since soft tissues often attach to bony prominences. Saigo et al. [26] defined the femoral sMCL attachment similarly and found it at 47% A-P and 48% P-D distance. However, they defined a 0% datum at the anterior femoral shaft and not at the anterior border of the MFC, so the results of the two studies are very much the same. In contrast, one study [20] described the centre of the femoral sMCL attachment 5 mm posterior and 3 mm proximal to the ME, which is outside the mean limit of the attachment area in the present study. The exact femoral attachment site is important: if a graft is placed posteriorly it will slacken with knee flexion [34], or could be too tight in extension. The centre of the femoral attachment of the sMCL is at the same distance from the distal and posterior surfaces of the medial femoral condyle, which suggests that a reconstruction placed on the ME will be isometric. Even small alterations in position can make a big difference to the performance of a construct so it is imperative during surgery to check for isometry of a proposed construct by connecting guide pins placed at the proposed attachment sites on femur and tibia with a suture and taking the knee from full extension to high flexion.

The ME is on a relatively flat area rather than being a single localised prominence and so locating it is not easy. Studies using surgical navigation systems have found much variability in the identification of the ME [15, 17, 29]: Jerosch et al. [15] reported that it was found across a range of 22 mm in one knee, with mean inter-observer error of 10 mm. Thus, a radiograph of a guidewire positioned by palpation may be very useful. The radiographic method of Schöttle et al. [27] was used by Wijdicks et al. [33] to obtain A-P measurements similar to those reported in the present study, but with P-D measurements differing markedly by 6–9 mm. This may reflect variations in identifying the most posterior/proximal point of Blumensaat’s line.

This study found mean inter-observer differences of ± 1.8 mm in A-P and ± 0.9 mm in P-D directions, so there is little point in describing an anatomical measurement for surgical guidance to less than the nearest whole mm. While previous quantitative reports of the medial knee anatomy have not performed a repeatability analysis for detecting the ME [20, 21, 26], future studies should ascertain and report their precision. This study, and others [33], found that radiographic measurements are accurately repeatable by different examiners and therefore could be a valuable tool for clinical practice. It is critical to obtain a perfect lateral radiographic projection to avoid error, and surgeons need discipline to not accept radiographic projections that are ‘close to acceptable’!

An important novel finding is that the dMCL is fan-shaped, oriented antero-distally towards a 22 mm wide tibial attachment. Analogous to the oblique fibres of the “anterolateral ligament”, the dMCL acts as an important restraint against tibial rotation [2, 4, 24]. Tibial external rotation tightens the dMCL rapidly [25, 34]. Thus, it is ideally oriented to resist external rotation and anterior translation of the medial tibial plateau—one could argue like an ‘anteromedial ligament’! Robinson et al. [25] described the dMCL tibial attachment as 10–13 mm wide and 2–3 mm distal to the articular cartilage margin, while Liu et al. [21] reported it as 6.5 mm below the joint line. Both studies located the centre of the dMCL femoral attachment posterodistal to the ME/sMCL. Although early techniques in ACL surgery treated anteromedial rotatory instability by a pes anserinus transfer [28], those non-anatomical techniques have lost popularity. As no dMCL reconstruction to restore rotatory stability has been described, the present findings provide basic knowledge for further development. Excessive anteromedial rotatory laxity may be left unaddressed by surgeons because they lack the necessary operative techniques, but unaddressed MCL laxity is associated with ACL graft failure [11, 30].

The PMC has an extensive femoral attachment extending posterior from the sMCL attachment around the medial femoral condyle distal to the adductor tubercle. The POL, a distinct band within the PMC, attaches at a mean of 11 mm posterior and 4 mm proximal to the ME. This position is similar to that found in a previous CT study [26]. The POL has also been located 8 mm distal and 6 mm posterior to the adductor tubercle [20].

The present study has some limitations. Some of the small number of specimens were older than typical of MCL injured patients. However, MCL anatomy has not been shown to change over lifetime. Gross inspection and radiographs precluded osteoarthritis and joint line narrowing in all specimens, and thus measurements relative to the joint line were not affected. Even though three investigators were blinded to the others’ results when they identified the ME, this is subjective and there might be a difference in the interpretation by other surgeons. A strength of this study is the analysis of the precision of measuring anatomical locations by both optical digitisation and radiography of fresh specimens.

This study has described the bone attachments of the sMCL, dMCL, and POL, and transferred the anatomical observations to clinically relevant localisation in true-lateral radiographs. These findings should help surgeons to identify landmarks and attachment points to anatomically reinsert an avulsed ligament, to accurately position drill holes for MCL reconstruction, or to critically assess graft positions at review. In addition the oblique orientation of the dMCL implies its importance in restraint of tibial external rotation. This is very relevant to anteromedial rotatory instability in cases combined with ACL rupture.

## Conclusion

The locations of the femoral and tibial attachments of the superficial and deep MCLs, and the POL, have been described in relation to overall knee sizes and also in relation to osseous landmarks, using both optical digitisation and radiography. The femoral sMCL attachment envelops the ME and has a long distal tibial attachment. The dMCL has an antero-distally oblique course as it fans out to a wide anteromedial tibial attachment. The POL attaches proximal and posterior to the femoral ME and inserts at the posteromedial tibial rim.
